# Effect of Tai Chi on mononuclear cell functions in patients with non-small cell lung cancer

**DOI:** 10.1186/s12906-015-0517-7

**Published:** 2015-02-05

**Authors:** Jing Liu, Peijie Chen, Ru Wang, Yonghong Yuan, Xueqiang Wang, Chunying Li

**Affiliations:** Department of Martial Arts, Shanghai University of Sport, Shanghai, 200438 China; Department of Psychology, School of Kinesiology, University of Michigan, Ann Arbor, MI 48109 USA; Department of Sports Medicine, Shanghai University of Sport, Shanghai, 200438 China; Department of Sports Leisure, Shanghai University of Sport, Shanghai, 200438 China; Department of Sport Rehabilitation, Shanghai University of Sport, Shanghai, China; Department of Biochemistry & Molecular Biology, Wayne State University School of Medicine, 540 E. Canfield, 5312 Scott, Detroit, Michigan 48201 USA

**Keywords:** Physical activity, Peripheral blood, Immune cell, Cell proliferation, Cytolytic activity

## Abstract

**Background:**

Tai Chi is the Chinese traditional medicine exercise for mind-body health. The objective of this study is to investigate the effect of Tai Chi Chuan (TCC) exercise on the proliferative and cytolytic/tumoricidal activities of peripheral blood mononuclear cells (PBMCs) in postsurgical non-small cell lung cancer (NSCLC) patients.

**Methods:**

Patients (n = 27) were randomly divided into the control group (n = 13) and the TCC group (n = 14). TCC group participated in Tai Chi 24-type exercise for 16 weeks, 60-min every time, and three times a week. Peripheral blood was collected and PBMCs isolated before and after the 16-week TCC, PBMC proliferation and co-culture of PBMCs with the NSCLC cell line A549 were performed for proliferation and cell cytolysis assays. Analysis of NKT cells, NK cells, and CD123+ and CD11c + dendritic cells were also performed.

**Results:**

(1) After 16-week of TCC, cell proliferation increased significantly as compared with the control. (2) PBMCs from the TCC group also demonstrated enhanced cytolytic/oncolytic activity against A549 cells. (3) Significant differences were also found in NK cell percentage at t = 16 weeks, post-pre changes of NKT and DC11c between groups.

**Conclusion:**

Regular Tai Chi exercise has the promise of enhancing PBMC proliferative and cytolytic activities in NSCLC patients. Our results affirm the value of a future trial with a larger scale and longer duration for cancer survivors.

**Trial registration:**

ChiCTR-TRC-11001404.

## Background

Lung cancer is still the deadliest cancer in the world, with approximately 80% of the cases being non-small cell lung cancer (NSCLC) [1]. Despite progress in the lung cancer treatment over the past two decades, 5-year survival rate following the conventional approaches still remains low, estimated in the range of 5-20% [2]. Clearly, there is still an unmet medical need for new alternative therapies that demonstrate efficacy in lung cancer treatment with less associated toxicity than chemotherapy.

Exercise has been shown to improve blood immune function in cancer survivors. The improvements that have been shown include increase in natural killer (NK) cell, cytotoxic activity, monocyte function, and the proportion of circulating granulocytes [3]. Tai Chi Chuan (TCC) is very popular as a traditional exercise in China. Tai Chi practitioners seek their body’s constant movement during Tai Chi exercise and must concentrate, breathe deep, and put aside distracting thoughts [4]. So, Tai Chi is commonly described as mind-body practice and recently has been evaluated as a possible therapeutic strategy or a complementary and alternative medicine for distinct health concerns [4]. Significant improvements have been reported in balance, cardiovascular fitness, aerobic capacity, muscular strength, blood pressure, and psychological well-being in those individuals practicing TCC, and Qi Gong, another form of mind–body traditional Chinese medicine also with a long history [5-8]. TCC practice in breast cancer survivors significantly enhanced functional capacity and health-related life quality, decreased fat mass with increased IL-6 and decreased IL-2 levels, and maintained insulin level compared to the breast cancer survivors only receiving psychosocial support [9].

We recently reported that a 16-week TCC intervention prevented the increase of circulating CD4+ T helper Type 2 (Th2) cells and CD8+ cytotoxic T cells, Type 2 (Tc2) levels, but not of Th1 and Tc1 levels, in NSCLC survivors [10]. However, little is known about the physiologic effects of TCC on immune function in NSCLC survivors. Yannelli *etc.* [11] reported that peripheral blood mononuclear cells (PBMCs) from NSCLC patients produced more cytokines and higher combined levels of Th1 and Th2 cytokines, with higher level of circulating Tregs and a reduced lymphoid proliferative response. PBMCs from lung cancer patients are clearly different from those obtained from normal donors, and likely reflect the influence of tumors *in vivo*.

In the present study, we reported the effects of Tai Chi exercise on changes in PBMC proliferative and cytolytic activities. We hypothesized that Tai Chi invention would increase PBMC proliferative and cytolytic activities in NSCLC patients.

## Methods

### Participants

This study was the further study following our previous report, in which the detailed methods of assessing the effect of TCC on main hormone levels (cortisol, catecholamine, β-endorphin), and cytokines (IFN-γ, IL-4) of Th1/Th2 and Tc1/Tc2 reaction have been described [10]. Participants were recruited at the Shanghai Lung Cancer Center. Approval from the institutional review board of Shanghai University of Sport was obtained before acquisition of written consent and enrollment of participants. Potential participants were required to meet the following criteria for inclusion in this study: (1) primary diagnosis of NSCLC stages I to IIIB, (2) the initial surgical resection was lobectomy (post-lobectomy lung cancer patients), (3) two or more years after completion of surgical intervention, (4) no habitual exercise activities, (5) absence of contraindications to supervised aerobic exercise training based on cardiopulmonary exercise testing, and (6) physically capable to participate in a physical activity regimen. The criteria for exclusion included: (1) autoimmune disorders treated with immunosuppressive drugs, (2) malignancies treated with chemotherapy, (3) other diseases treated with corticosteroids and/or nonsteroid anti-inflammatory drugs.

### Study design

**Figure 1:**
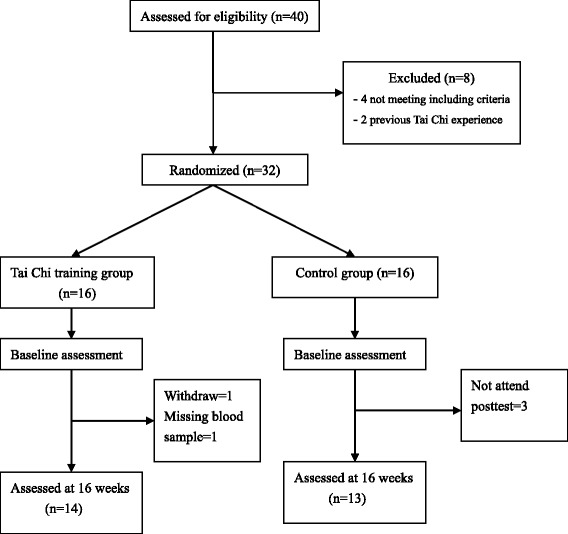
**Study flow CONSORT diagram.** Subjects (n = 32) were randomly assigned to 2 groups, the control group (n = 16) and the Tai Chi Chuan (Tai Chi) group (n = 16). The total number of subjects who participated in end-point measurements was 27 (82%), 13 subjects in the control group and 14 subjects in the Tai Chi group. Reasons for not completing the intervention included withdraw (n = 1), missing blood sample (n = 1), and not attend posttest (n = 3).

We designed a randomized trial to investigate the effect of 16 weeks of Tai Chi exercise in a group of lung cancer patients. Participants were randomly assigned into the control group (n = 16) and the TCC group (n = 16) using numbered envelopes into which a card indicating patient allocation had been placed according to a computer-generated random-number sequence. Adherence and compliance in the trial were monitored through attendance records and personal records kept by each participant. Twenty-seven participants, 13 in the control group and 14 in the TCC group completed the study (Figure 1). The control group consisted of 7 males and 6 females with a mean age of 60.46 ± 7.08 years. The TCC group included 8 males and 6 females with a mean age of 62.64 ± 8.35 years (Table 1). The TCC group was led by an expert Tai Chi practitioner with more than 20 year experience to practice TCC for 16 weeks. Both groups received hospital care as scheduled, and the control group only received hospital care. Neither of the 2 groups had taken any Chinese herbal medicine in their post cancer phase and during the study period.

**Table 1:** **Demographic and clinical characteristics of the study participants**

**Variable**	**TCC group**	**CON group**
	**(n = 14)**	**(n = 13)**
Age, yrs	62.64 ± 8.35	60.46 ± 7.08
Weight, kg	65.46 ± 7.69	65.63 ± 8.35
Body mass index, kg/m^2^	21.85 ± 1.79	22.13 ± 2.06
Female, No. (%)	6(43%)	6(46%)
Duration of disease, yr	6.14 ± 5.35	5.31 ± 4.16
Years Postsurgery, yr	5.74 ± 5.63	5.2 ± 4.12
Stage I/II/III, No.	3/5/6	4/5/4
Disease category:		
Squamous	7(50%)	6(46%)
Adeno	5(36%)	5(38%)
Bronchoalveolar	2(14%)	1(8%)
Adeno-squamous	0(0%)	1(8%)

### TCC exercise intervention

The goal of the trial was to test the effect of Tai Chi exercise on the immune functions of post-lobectomy surgery NSCLC survivors. The protocol of Tai Chi was specially designed to regulate consciousness, train breath and improve physical fitness by practicing Tai Chi static relaxation, breathing gently and deeply, and performing symmetric movements. The exercise for the first 8 weeks emphasized static relaxation methods with music, breathing with up-down and open-close movements and the mastery of single forms through multiple repetitions; exercise in later 8 weeks focused on 24-form Tai Chi based on Yang style and enhancing coordination of mind relaxation, breathing and locomotion. Each TCC session lasted for 60 minutes with a 15-minute warm-up period, a 35-minute practice period, and a 10-minute cool-down period (i.e., breathing with gentle stretching and tapping or self-massage). During the 16-week study, participants in the TCC group practiced TCC 3 times per week for at least 60 minutes each time from 6:30 am to 7:30 am at a community center.

### Blood sampling

Peripheral venous blood was collected from all participants at approximately the same time of the day (7:00 am) before (t = 0) and after 16-week (t = 16) TCC exercise. Blood samples were analyzed within 24 hours of collection. On the days of blood collection, subjects did not carry out any TCC exercise. All subjects refrained from any exercise for at least 24 hours before the blood collection/sampling.

### PBMC proliferation and cytotoxicity analysis

PBMCs were isolated by density gradient centrifugation using Ficoll-Hypaque (Amersham Pharmacia, China) according to the manufacturer’s protocol. The freshly isolated PBMCs were used for the proliferation assay and cytotoxicity assay. Proliferation assay was performed in 96-well plates at cell density of 0.5 × (10^6) and 1 × (10^6) using MTT cell proliferation kit (ATCC, USA), and the optical density (OD) was read at a wavelength of 570 nm in a microplate reader as reported before [12].

PBMC cytotoxicity assay was performed by co-culture of PBMCs from the participants with a human NSCLC cell line A549. A549 cells were seeded in 96-well flat-bottom plates at a density of 5 × 10^3/well and left for several hours to adhere. The cytotoxicity of freshly isolated PBMCs from both groups was tested in triplicate against tumor cells (A549) at various effector/target (E:T) cell ratios (12.5:1, 25:1, and 50:1) after 24 h of co-culture. The tumor cell (A549) viability was determined by Cell Counting Kit-8 (Dojindo Molecular Technologies, Japan) according to the manufacturer’s instructions and absorbance (OD value) at 450 nm was measured spectrophotometrically using a microplate reader as previously reported [13].

### Analysis of T cell subset, NKT cells, and NK cells

Flow cytometric analysis for NK/NKT cells was performed as reported before [14]. Peripheral blood (100 μl) was incubated with anti-human CD4-fluorescein isothiocyanate (FITC), anti-human CD8-phycoerythrin (PE), as well as anti-human CD3-peridinin chlorophyll protein (PerCP)-Cy5 antibodies (Immunotech Co). Thereafter, CD3+, CD4+, and CD8+ T cells were measured using fluorescence detection by flow cytometry. Peripheral blood was stained with anti-human CD3-FITC, anti-human CD16-PE, and anti-human CD56-PE antibodies (Beckman Coulter) before subjected to flow cytometry. NK cells were defined as CD3–/CD16+/CD56+ cells, and NKT as CD3+/CD16+/CD56+ cells [15].

### Analysis of CD123+ and CD11c + dendritic cells (DCs) by direct immunofluorescence staining of whole blood

Monoclonal antibodies were used as described before [14]. Peripheral blood cells were analyzed by three-color flow cytometry. Briefly, aliquots (100 μl) of peripheral blood were incubated with a mixture of FITC-conjugated anti-lin mAbs (BD Biosciences), a PerCP-conjugated anti-HLA-DR mAb, and either a PE-conjugated anti-CD11c mAb to detect myeloid DC (mDC) or a PE-conjugated anti-CD123 mAb to detect plasmacytoid DC (pDC), or a PE-conjugated isotype control as we reported before [14]. All incubations were performed at room temperature in the dark. DCs were defined as the cells positive for PerCP-conjugated anti-HLA-DR mAb and negative for FITC-conjugated lin 1. Anti-CD11c or anti-CD123 mAb conjugated with PE was used for further identification of the mDC and pDC subsets.

### Statistical analyses

**Figure 2:**
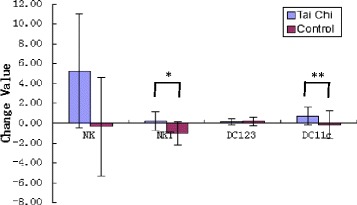
**Changes in mean NK, NKT, and DC cells in the Tai Chi group and the control group.** The units of the change values (NK, NKT, DC123 and DC11c) are percentage of cells in CD3+ lymphocytes. The error bars represent SD of change values. * p < 0.05; ** p < 0.01 compared between TCC group and control group.

All data were checked for normality using the Shapiro-Wilk W test in SPSS (version 15.0, for Windows). If data were not normally distributed, a natural logarithm transformation was applied. A sample sizes of 16 (8 subjects per group) achieved 80% power to detect an effect size of 0.626 using an F test with a 0.05 significance level. Data are presented as mean ± standard deviation, and independent (unpaired) samples *t*-test was used to assess differences between control and TCC group before and after intervention. Paired-samples *t*-test was also used to assess differences between variables pre- and post-intervention. In addition, for immune cells of each individual, pre-test values were subtracted from post-test values to obtain individual change values (see Figure 2). When outcome variables showed significant differences in group-by-time interaction, absolute change values were compared between groups using independent t tests. *p* < 0.05 was considered statistically significant.

## Results

### Baseline characteristics

Table 1 presents baseline characteristics. No significant differences were observed between groups for age, weight, body mass index, disease duration, length of post-surgery, and lung cancer category.

### Change in PBMC proliferation

Table 2 shows the change in PBMC proliferation. The significant changes of PBMC proliferative capacity were observed in TCC group at t = 16 weeks compared to t = 0 week, OD (indicating cell proliferation) increasing from 0.70 ± 0.28 to 0.92 ± 0.24 (*p* < 0.05) at 0.5 × (10^6) cell density, and from 1.21 ± 0.23 to 1.66 ± 0.37 (*p* < 0.001) at 1 × (10^6) cell density; whereas PBMCs from control group did not show any significant difference in proliferation: 0.89 ± 0.26 to 0.99 ± 0.25 (*p* = 0.284) at 0.5 × (10^6) cell density and 1.33 ± 0.26 to 1.39 ± 0.18 (*P* = 0.537) at 1 × (10^6) cell density. However, an independent (unpaired) samples t-test had failed to detect any significant difference in PBMC proliferation between the two groups, even though TCC group demonstrated higher cell proliferation (1.66 ± 0.37) compared to the control group (1.39 ± 0.18) at 1 × (10^6) cell density.

**Table 2:** **Effect of TCC on PBMCs proliferation, cytotoxic activity and immune cells – comparisons between control group at t = 0 and at t = 16 week, and between TCC group at t = 0 and at t = 16 week**

	**Control**		**TCC**	
	**t = 0**	**t = 16 weeks**	**t = 0**	**t = 16 weeks**
**PBMCs proliferation (OD at 570 nm)**				
0.5 × 10^6^	0.89 ± 0.26	0.99 ± 0.25	0.70 ± 0.28	0.92 ± 0.24*
1 × 10^6^	1.33 ± 0.26	1.39 ± 0.18	1.21 ± 0.23	1.66 ± 0.37‡
**PBMCs cytolytic activity (A549 viability; OD at 450 nm)**				
50:1 E:T ratio	1.04 ± 0.33	0.99 ± 0.25	1.04 ± 0.19	0.51 ± 0.15‡^▲^
25:1 E:T ratio	0.85 ± 0.39	0.76 ± 0.22	0.85 ± 0.16	0.51 ± 0.13‡^▲^
12.5:1 E:T ratio	0.66 ± 0.20	0.63 ± 0.20	0.53 ± 0.15	0.52 ± 0.20
**Immune cells**				
NK (%)	20.49 ± 6.82	20.17 ± 7.35	22.66 ± 8.23	27.94 ± 10.3^▲^
NKT (%)	3.89 ± 2.59	2.87 ± 1.71	3.58 ± 2.97	3.83 ± 3.03
DC11c^+^ DCs (%)	1.63 ± 0.80	1.47 ± 0.61	2.37 ± 1	3.09 ± 1.57
DC123^+^ DCs (%)	0.42 ± 0.22	0.63 ± 0.38	0.95 ± 0.44	1.10 ± 0.58

Values are expressed as mean ± SD. Significant differences between t = 0 week and t = 16 week in control group as well as in TCC group are indicated as follows: *p < 0.05; ‡*p* < 0.001, using paired-samples *t*-test. Significant differences between TCC group and control group at t = 16 week are indicated as ^▲^ *p* < 0.05, using independent samples *t*-test.

### Change in PBMC cytolytic activities

Table 2 shows the change in PBMC cytolytic/oncolytic activity against lung cancer cells A549. Cancer cell A549 viability was assessed to reflect the cytotoxicity of PBMCs from both groups. Tai Chi exercise induced a significant increase of cytotoxicity of PBMCs, as indicated by the profound decrease of A549 viability in TCC group at t = 16 weeks compared to t = 0 week, OD decreasing from 1.04 ± 0.19 to 0.51 ± 0.15 (*p* < 0.001) at 50:1 effector-to-target cell ratio, from 0.85 ± 0.16 to 0.51 ± 0.13 (*p* < 0.001) at 25:1 effector-to-target cell ratio, but no significant difference at 12.5:1 effector-to-target cell ratio. However, PBMCs from control group did not show any significant difference in their tumor killing capacity. We also performed an independent (unpaired) samples t-test and our result showed that cytotoxicity of PBMCs was significantly increased following Tai Chi exercise (t = 16 weeks) in TCC group as compared to that in control group when assessed at 25:1 and 50:1 effector-to-target cell ratio (*p* < 0.05) (Table 2).

### Changes in immune cells

Table 2 shows the change in percentages of NK, NKT, CD123+ and CD11c + dendritic cells in peripheral blood. While no significant changes were observed for most of the parameters in both groups at t = 16 weeks compared to t = 0 week, significant differences were found in percentage of NK cells (*p* < 0.05) between two groups at t = 16 weeks (Table 2). In addition, significant differences were also observed for post-pre changes of NKT (*p* < 0.05) and DC11c (*p* < 0.01) between groups (Figure 2).

## Discussion

To date, there have been no standard rehabilitation options for those postsurgical NSCLC patients with multiple comorbid conditions. In the current study, we performed immunologic analysis on PBMCs obtained from postsurgical Chinese NSCLC survivors who were randomized to a TCC intervention group and a control group. Our results revealed that a 16-week, moderate-intensity TCC program significantly promoted PBMC proliferation and cytotoxicity against NSCLC tumor cells. However, there was no significant difference in percentage of NKT, CD123+ and CD11c + dendritic cells between the two groups, with the exception of NK cells at t = 16 weeks that demonstrated a significant increase in TCC group as compared to control group.

Recent researches reported an effective role of exercise in improving VO_2_ peak, pulmonary and skeletal muscle functions, and life quality, as well as ameliorating fatigue and depression in NSCLC individuals [16,17]. Moreover, there is an increasing amount of evidence revealing a connection between physical exercise and cancer survival, probably through modulating/improving immune function in cancer survivors [9,18,19]. TCC, an integrative medicine mind-body practice, elicited similar beneficial effects to breast cancer survivor as more traditional exercise programs, significantly enhancing functional capacity, and the maintenance of insulin levels compared to a population of breast cancer survivor only receiving psychosocial support [9,20]. In one of our previous studies, we reported that TCC promotes the development of Th1 immune responses associated with the immune modulation of NKT and DCs in middle-aged and elderly women after completion of a 6-month TCC exercise [14]. However, research on TCC intervention on cancers is still very limited, especially the effects of TCC on immune function modulation in those patients.

In the present study, we observed that, out of the 4 parameters of immune cells we tested in the NSCLC survivors, only NK cell percentage was significantly improved by 16-week TCC exercise. A 16-week intervention may not be long enough to see any significant changes in NKT and DC cells. NK cells and NKT cells kill infected cells and also tumor cells, and play a pivotal role in providing signals to initiate the adaptive immune response. It was reported that NK cells were affected by soluble factors that are associated with tumor growth [21], and as tumors progress NK cell numbers decreased [22]. It was speculated that physical activity could improve the number and/or function of NK cells, which have a positive role in tumor suppression [23]. Moyna *et al.* found that NK cells increased throughout 18-min incremental cycling exercise in healthy subjects [24]. Our recent study showed that TCC exercise increased the percentage of NK cells in middle-aged and elderly women [14]. Here, we observed a similar beneficial effect of TCC in augmenting the percentage of NK cells in TCC group relative to control group in NSCLC survivors.

NKT cells contribute to tumor immunosurveillance via endogenous IL-12 pathway. Numerous studies reported cancer-related type-I NKT cell defects in various types of human cancers, including advanced prostate cancer, multiple myeloma, melanoma, and colon, lung, and breast cancers [25]. It was shown that DCs produce high levels of co-stimulus molecules to activate T cells, after combining DCs and T cells to mediate the Th1 response by secreting large amounts of IL-12, helping to eliminate tumors [26]. Hayes *et al.* [27] found no change in the speed of immune cell recovery following a 12-week exercise intervention; however, the exercise program did not negatively impact immune function, either. The effects of TCC exercise on NK, NKT and DCs in our current study demonstrated that 16-week TCC may reverse the lymphocyte number change trend in NSCLC survivors. However, longer-term follow up research in those cancer survivors is needed to draw any meaningful conclusion.

In our present study, we also observed that TCC exercise significantly improved PBMC proliferative and cytolytic activities. By applying an independent (unpaired) samples t-test we found that cytotoxicity of PBMCs was significantly increased following Tai Chi exercise (t = 16 weeks) in TCC group as compared to that in control group when assessed at 25:1 and 50:1 effector-to-target cell ratio (p < 0.05) (Table 2). However, an independent samples t-test failed to detect any significant difference in PBMC proliferation between the two groups, even TCC group demonstrated higher cell proliferation (1.66 ± 0.37) compared to the control group (1.39 ± 0.18) at 1 × (10^6) cell density. We speculate that it may be due to the small sample size and/or short intervention time. PBMCs consist of lymphocytes, monocytes, and macrophages, etc., and they are critical components in the immune system to fight infections. Studies have shown a significant reduction of peripheral blood natural cytotoxicity in patients with a wide variety of cancers, compared with non cancer-bearing controls [28-30]. Other authors, however, have documented no reduction in natural cytotoxicity in patients with colorectal and breast cancers, irrespective of the stage of disease [31,32]. Natural cytotoxicity, mediated by NK cells, is believed to play an important role in host anti-cancer defense mechanisms. It was reported that moderate exercise had a beneficial effect on the function of *in vitro* NK cells in stomach cancer patients after curative surgery [18], and also in women with breast carcinomas [33]. A comprehensive literature search also revealed that physical exercise improves immune system function including NK cytotoxic activity, monocyte function and proportion of circulating granulocytes in various cancer survivors [3]. Increased NK cytotoxicity may be due to the increased number of NK cells in the circulation. However, Fairey *et al.* showed that 15-week cycle ergometer exercise improved NK cytotoxic activity on a single cell basis in breast cancer survivors [34]. These aforementioned studies, along with the findings from our present study, suggest that TCC have potential to stimulate PBMC cytotoxicity and improve anti-tumor cellular function.

Inconsistent results have been reported with respect to the effect of physical exercise on PBMC proliferation. Some studies have reported that regular moderate exercise increased the antigen-induced T cells proliferation [35]. It was reported that cycle ergometer exercise increased unstimulated lymphocyte proliferation in breast cancer survivors [33]. In the present study we observed an enhanced PBMC proliferation following 16-week TCC in NSCLC patients. However, some studies reported a decrease [36,37], or no effect [27,38] of regular, moderate exercise on T cell proliferation response to mitogens or unstimulated lymphocyte proliferation in both humans and experimental animals. Variability in the reports examining the relationship between exercise and PBMC cellular function may be attributed to both host factors (such as training status) and the nature of the exercise paradigm.

### Study limitations

Given the pilot nature of this study, its relatively small size, lack of an active control group, and the complexity of the intervention, the current study has several limitations that need to be addressed. First, this study was not blinded or placebo controlled, thus it is possible that the benefits reported from the TCC intervention were due to participant bias or other non-specific effects (e.g., differences in patient attention or social interaction). Second, the relatively small sample size cannot rule out the possibility that the findings from this study were due to chance, instead of the TCC intervention. Third, the 16-week duration of the study is relatively short in regard to providing an adequate dose of TCC intervention and observing significant changes in other parameters/biomarkers. Fourth, the use of homogeneous lung cancer survivors alone limits the generalizability of these results to a more broad population of cancer survivors (e.g., young population, other types of cancer survivors, individuals undergoing chemotherapy treatments). Lastly, due to the voluntary nature of this study, subjects who chose to participate may have been particularly receptive to TCC exercise, and the results may not be generalizable to those less amenable to this mode of exercise. Therefore, further studies are needed to confirm our findings in larger sample size of lung cancer survivors and investigate the changes of different biomarkers by altering Tai Chi parameters and intervention period. In addition, combining different exercise regimens (such as Tai Chi, Qigong, and acupuncture) [39-42] may have synergistic or additive effects on the health and life quality of cancer survivors, representing a very promising complementary and alternative medicine for cancer patients.

## Conclusions

The results from the current study suggest that regular Tai Chi exercise has the potential of significantly enhancing PBMC proliferative and cytolytic activities in NSCLC patients. Future experiments need to explore the precise effects of Tai Chi exercise on tumor-specific immunity in view of molecular mechanisms and mind-body control. Additional research is also needed to determine if Tai Chi exercise in cancer survivors may reduce the risk of cancer recurrence and secondary malignancies and increase survival.

## Abbreviations

TCC: Tai Chi Chuan
PBMC: Peripheral blood mononuclear cell
NSCLC: Non-small cell lung cancer
OD: Optical density
E:T: Effector/target
FITC: Fluorescein isothiocyanate
PE: Phycoerythrin
PerCP: Eridinin chlorophyll protein
